# Investigation of Surface Modification Effects on the Optical and Electrical Hydrogen Sensing Characteristics of WO_3_ Films

**DOI:** 10.3390/s25237268

**Published:** 2025-11-28

**Authors:** Jiabin Hu, Jie Wei, Jianmin Ye, Wen Ye, Ying Li, Zhe Lv, Meng Zhao

**Affiliations:** 1 School of Physical Science and Technology, Suzhou University of Science and Technology, Suzhou 215000, China; 2 School of Information and Communication, Harbin Institute of Technology, Harbin 150001, China; 3 Advanced Microscopy and Instrumentation Research Center, Harbin Institute of Technology, Harbin 150080, China; 4 School of Electronic and Information Engineering, Suzhou University of Science and Technology, Suzhou 215000, China

**Keywords:** hydrogen sensor, tungsten oxide, surface modification, optical and electrical responses

## Abstract

The development of hydrogen energy is advancing rapidly, while the progress of hydrogen sensors has been relatively lagging behind and cannot meet the stringent performance requirements of hydrogen energy applications. WO_3_ has attracted significant attention due to its highly complementary optical and electrical responses to hydrogen. In this study, hydrogen-sensitive WO_3_ thin films characterized by vertically aligned crystallites were fabricated by modulating the substrate temperature and oxygen pressure during pulsed laser deposition. Building upon this foundation, a comprehensive investigation into surface modification strategies for enhancing sensitivity was undertaken. The surface modifications encompassed eight distinct metals and four different metal oxides. Among the metal-modified samples, palladium (Pd) Pd exhibited a markedly enhanced electrical response, defined as the ratio of the resistance in hydrogen-free air to that in hydrogen, of 1022, corresponding to ~45 times higher than the value of 22.4 achieved for Pt-modified films and 120 times higher than the value of 8.4 for Au-modified films. In addition, Pd/WO_3_ films showed a measurable optical transmittance change of 9.7%, while all other metal-modified samples exhibited negligible optical responses (<1%). This enhancement is attributable to the catalytic and electronic sensitisation effects of Pd. Conversely, metals such as platinum (Pt), gold (Au), and silver (Ag) elicited negligible optical responses, suggesting minimal catalytic activity. The electrical response in these cases was primarily governed by electronic sensitization effects related to the work function of the metal, with higher work function values correlating with more pronounced sensitization. Regarding metal oxide modifications, the sensitization effect was more substantial when the disparity in work function between the oxide and WO_3_ was greater, and this enhancement was found to be independent of the charge carrier type of the modifying oxide. These results offer significant insights into the design principles underlying high-performance WO_3_-based hydrogen sensors and underscore the pivotal influence of surface modification in governing their sensing characteristics.

## 1. Introduction

Hydrogen represents a sustainable, clean, and renewable energy resource. Hydrogen fuel cell vehicles provide several benefits, including zero emissions, extended driving ranges, and rapid refueling times [1]. In recent years, national policies and financial support from the United States, China, and other countries have driven substantial advancements in hydrogen-related technologies, including hydrogen production by water electrolysis [2] and hydrogen fuel cells [3], which have even enabled the commercial operation of hydrogen fuel-cell buses. In all of these hydrogen-related technologies, reliable hydrogen sensors are essential components that continuously monitor possible leaks, trigger safety shutdowns or ventilation, and prevent the formation of flammable or explosive H_2_-air mixtures. Nevertheless, conventional hydrogen sensors fail to satisfy the rigorous technical demands of emerging applications, including hydrogen fuel cell vehicles, hydrogen refueling stations, and high-pressure hydrogen storage systems, thereby posing significant safety concerns [4,5,6]. According to the guidelines of the U.S. Department of Energy for on-board hydrogen detection [5], hydrogen sensors should achieve a measurement range of 0.1–10 vol % H_2_, operate reliably between −30 °C and 80 °C, exhibit a response time shorter than 1 s, and maintain a service lifetime exceeding 10 years. However, most existing hydrogen sensors fail to simultaneously satisfy these requirements, particularly in terms of fast response and long-term stability.

In contrast to most n-type hydrogen-sensitive oxide materials such as ZnO and SnO_2_, WO_3_ not only exhibits a decrease in electrical resistance exceeding three orders of magnitude, but also demonstrates strong absorption in the red and near-infrared spectral regions, leading to a characteristic blue coloration known as the gasochromic effect [7,8,9]. The gasochromic properties of WO_3_ provide a foundation for the development of an optical sensing mechanism, as hydrogen-induced variations in color and transmittance can be detected through the use of a laser and photodiode. Extensive research has indicated that the electrical response of WO_3_ is highly sensitive to low hydrogen concentrations (1 ppm) but tends to saturate at higher levels (≥1% *v*/*v*) because most surface adsorption sites become occupied, leading to a plateau in resistance change [10,11,12], whereas its optical response allows quantitative differentiation of hydrogen concentrations—particularly at elevated levels (100 ppm—100%)—although the dependence is not strictly linear [13,14,15,16,17]. By integrating the electrical signal, which is effective for detecting low hydrogen concentrations, with the optical signal, which is suitable for high-concentration detection, the development of a photoelectrically coupled hydrogen sensor based on WO_3_ thin films shows considerable potential to substantially broaden the detection range, enabling comprehensive monitoring across a wide spectrum of hydrogen concentrations.

WO_3_ thin films have been fabricated by various physical vapor deposition techniques such as pulsed laser deposition (PLD) [18,19], magnetron sputtering [20,21], and thermal evaporation [22,23,24], each enabling distinct control over film stoichiometry, crystallinity, and morphology. PLD offers a wide controllable range of deposition parameters (e.g., laser fluence, substrate temperature and oxygen partial pressure). Among them, the oxygen partial pressure allows precise modulation of the energy and oxidation states of the deposited species, thereby tuning defect density, carrier concentration and surface chemisorption sites that directly govern gas sensing performance.

With respect to surface modification aimed at enhancing sensitivity, prevalent strategies include the formation of Schottky junctions via noble metal catalysts and the construction of heterojunctions composed of different oxide materials [25,26,27,28]. Previous studies have investigated the hydrogen sensing behavior of WO_3_ modified or doped with noble metals such as Pt [19], Pd [22,23], and Ag [21], as well as hybrid structures like WO_3_–ZnO and WO_3_–SnO_2_ composites. However, because the fabrication parameters of both the WO_3_ base films and the modifying catalysts differ greatly among these reports, their results are difficult to compare directly.

To address this, our study first optimized the PLD parameters to prepare WO_3_ films with a vertically aligned crystalline structure, which is favorable for gas diffusion and provides a reproducible platform for comparative surface modification. Based on this well-defined structure, we systematically investigated twelve different metallic and oxide modifiers under identical testing conditions. This approach enables direct evaluation of both electronic and catalytic sensitization effects and allows the identification of Pd and Pt as optimal modifiers for distinct sensing scenarios. In addition, the optical response was used as a criterion to qualitatively distinguish between the catalytic and electronic sensitization contributions of different surface modifiers.

In this study, PLD technology was employed to regulate the grain size, surface morphology, and stoichiometry of hydrogen-sensitive WO_3_ thin films. PLD was selected due to its significantly broader controllable range of oxygen partial pressure (0.001–1000 Pa) compared to other physical vapor deposition techniques such as sputtering (0.1–10 Pa) and evaporation (<0.001 Pa) [18,19,20,21,22,23,24]. This broad tunability enables precise control of stoichiometry and morphology during WO_3_ film growth. Although previous studies have reported WO_3_ films prepared by PLD, sputtering, or evaporation for hydrogen sensing, these investigations were generally limited in scope and lacked systematic optimization. In this work, we optimized the PLD parameters to prepare WO_3_ films with a vertically aligned crystalline structure that facilitates gas diffusion and provides a reproducible template for comparative surface modification studies. This approach allows direct evaluation of the influence of different surface modifiers on both optical and electrical hydrogen sensing responses.

The enhancement mechanisms associated with surface modification are generally explained by two theoretical frameworks: electronic sensitization and catalytic sensitization [27,28,29,30]. Electronic sensitization pertains to the modulation of hydrogen sensitivity through Schottky and heterojunction effects, whereas catalytic sensitization emphasizes the role of adsorption reactions between sensitizing materials and gas molecules. Despite extensive literature on metal modification mechanisms, there remains a paucity of studies that qualitatively or quantitatively differentiate between these two effects. To address this gap, the present work systematically investigates eight distinct metals and four metal oxides, categorized according to their work functions and electronic properties.

The first group of metals comprises Pd, Au, and Pt, with the corresponding work functions of 5.12 eV, 5.10 eV, and 5.65 eV [31]. Pd and Pt are well-known hydrogen catalysts, whereas the catalytic activity of Au in this context is less documented [27]. Given the similarity in work functions between Au and Pd, comparative analysis of their sensitization effects allows direct discrimination between electronic and catalytic contributions, while the larger work function disparity between Pd and Pt provides complementary insights [31].

The second group includes Ti (4.33 eV), Al (4.28 eV), Nb (4.3 eV), Ag (4.26 eV), and Ta (4.25 eV), all possessing work functions substantially lower than that of WO_3_ (5.17 eV) [31,32]. These metals were selected to compare their catalytic sensitization effects under similar electronic conditions and to distinguish catalytic contributions from electronic sensitization. For oxide-based heterojunction studies, n-type oxides Nb_2_O_5_ (4.85 eV) [33] and ZnO (5.21 eV) [34], as well as p-type oxides CuO (4.7 eV) [35] and NiO (5.26 eV) [36], were employed to modify the surface of WO_3_ thin films [37]. This systematic selection enabled the formation of n–n and p–n heterojunction configurations, facilitating qualitative evaluation of how work function disparity and junction type influence hydrogen sensing performance.

## 2. Experimental

### 2.1. Fabrication of WO_3_ Thin Films

**Figure 1:**
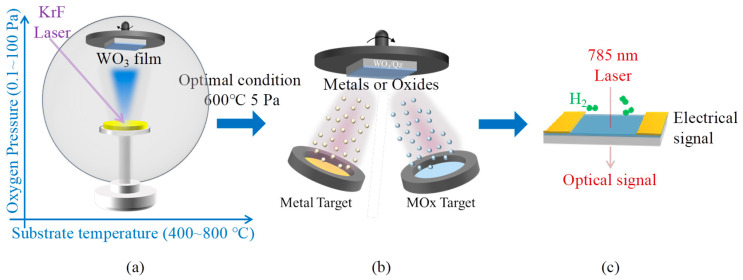
Research methodology for deposition and surface modification of WO_3_ films to enhance its optical and electrical hydrogen sensing properties. (**a**) Film depositioin; (**b**) surface decoration; and (**c**) hydrogen sensing test.

Figure 1 illustrates the sample preparation and testing procedures employed in this study. Initially, WO_3_ hydrogen-sensitive thin films were fabricated using PLD, Figure 1a. To determine the optimal conditions for WO_3_ film preparation, substrate temperature (ranging from 400 to 800 °C) and oxygen partial pressure during deposition (0.1 to 100 Pa) were systematically varied as control parameters. The influence of these preparation variables on the surface morphology, crystallinity, and visual characteristics of the WO_3_ films was thoroughly examined. Based on these findings, the deposition parameters most suitable for subsequent surface modification investigations were selected. The deposition process utilized a P180 pulsed laser deposition system (Necera, Huston, TX, USA), equipped with a 248 nm KrF excimer laser operating at a laser output power of 170 mJ, and a repetition rate of 1 Hz. The target material consisted of a 99.99% pure WO_3_ solid target supplied by MTI Company (MTI, Hefei, China), while the substrates were high-purity fused quartz amorphous substrates obtained from the same manufacturer.

### 2.2. Synthesis of Materials for Surface Modification

As illustrated in Figure 1b, the surface modification materials were fabricated utilizing a magnetron sputtering system (AdNanotek, Taiwan, China). In this study, two n-type MO_x_ semiconductors (Nb_2_O_5_ and ZnO) and two p-type oxide semiconductors (CuO and NiO) were selected to modify the surface of WO_3_ thin films, each with a nominal thickness of 3 nm, in order to investigate the regulatory influence of oxide semiconductor heterojunctions on the hydrogen sensing performance of WO_3_. All target materials, procured from MTI Company (China), possessed a purity ≥ 99.9%. To facilitate lateral comparison, the nominal thickness of the eight selected metals and four MO_x_ surface modification layers was uniformly maintained at 3 nm. Insufficient thickness of the modification layer would fail to achieve effective electron or catalytic sensitization, whereas excessive thickness would entirely obscure the WO_3_ surface [38]. Consequently, to precisely control the deposition thickness, extensive experiments were conducted to regulate the deposition rates of all materials within the range of 2 to 6 nm/min. Detailed preparation parameters and deposition rates for the twelve surface modification materials are provided in Supplementary Tables S1 and S2. It is noteworthy that metal modifications were deposited using a DC sputtering power supply with high-purity argon as the working gas, while MO_x_ modifications employed an RF sputtering power supply with a mixed oxygen-argon atmosphere. Additionally, in all experimental procedures, WO_3_ films as-prepared in the pulsed laser deposition system, were immediately transferred to the magnetron sputtering system. Upon achieving a base vacuum below 5 × 10^−6^ mbar, the samples were heated to 120 °C under vacuum conditions to desorb surface-adsorbed water, a critical step for forming high-quality heterojunction structures. Prior to deposition, samples were cooled to ambient temperature to ensure uniform deposition conditions. Post-deposition, all samples were stored in vacuum containers to preserve surface integrity and prevent oxidation during extended storage periods.

### 2.3. Structure Characterizations

The structural properties of the synthesized WO_3_ were comprehensively examined by employing a suite of complementary analytical methods. Crystalline phase identification was carried out via grazing-incidence X-ray diffraction (GI-XRD; Bruker, D8 Advance, USA) with Cu Kα radiation (λ = 1.5406 Å) at an incident angle of 0.5°. Single-crystal Si substrates with a 2–5 nm native oxide layer were used to avoid interference from the amorphous halo typically observed with fused-silica substrates, and no subtraction of amorphous background was applied. Nanoscale crystallographic details were elucidated through transmission electron microscopy (TEM; JEM-2100F, JEOL, Tokyo, Japan), which corroborated the lattice parameters determined by XRD through atomic-scale imaging. Surface morphology was characterized by field-emission scanning electron microscopy (FE-SEM; Gemini 300, ZEISS, Jena, Germany) and atomic force microscopy (AFM; Multimode VIII, Bruker, Billerica, MA, USA) operated in tapping mode. Additionally, measurements of film thickness were performed using a stylus profilometer (Dektak XT, Bruker, Billerica, MA, USA) applying a tip force of 3 mg. X-ray photoelectron spectroscopy (XPS) was conducted using a Thermo Fisher Scientific spectrometer equipped with a monochromatic Al Kα (hν = 1486.6 eV) radiation source. To prevent oxidation or contamination of oxygen-deficient samples during transfer, the surfaces were etched in situ under high vacuum using an Ar^+^ ion beam (etching area: 2 × 2 mm^2^; etching rate: 0.4 nm s^−1^; depth: 2 nm; voltage: 2 kV; emission current: 10 mA) prior to data acquisition. All spectra were calibrated against the C 1 s binding energy at 284.8 eV to correct for possible charging effects.

### 2.4. Evaluation of Hydrogen Sensing Characteristics

Figure 1c illustrates the methodologies employed for evaluating the optical and electrical hydrogen sensing performance of the sample. The hydrogen sensor performance was assessed using a high-precision automated dual-mode gas sensor signal measurement system developed by us [39]. A 2% *v/v* H_2_/air gas mixture was prepared by combining high-purity synthetic air with pure hydrogen. To facilitate simultaneous measurement of the optical and electrical responses of the sample, a six-way vacuum chamber equipped with high-transmittance quartz flanges at both the top and bottom was utilized. A photodiode was positioned beneath the bottom flange for transmittance measurements. Both the top quartz flange and the photodiode were fitted with bandpass filters to minimize interference from ambient light. A stable semiconductor laser with a wavelength of 785 nm served as the light source. The two electrodes were connected to a 6517B high-resistance meter (Keithley, Cleveland, OH, USA), which supplied voltage and concurrently measured current to determine resistance values. The sample testing temperature was maintained at 80 °C. This temperature was selected primarily to balance low power consumption and sensor selectivity, and secondarily to maintain the sensor temperature slightly above ambient conditions, thereby mitigating the influence of environmental temperature and humidity fluctuations [40].

In this study, the electronic response (S_e_) of the sensor is defined as the relative change in resistance, expressed by the equation, S_e_ = R_air_/R_H2_, where R_air_ denotes the baseline resistance in a hydrogen-free environment, and R_H2_ represents the stabilized resistance in an environment containing hydrogen at a specified concentration. The optical response (S_O_) is evaluated using the formula S_O_ = T_air_ − T_H2_, where T_air_ is the baseline transmittance of the film sample in air, and T_H2_ corresponds to the transmittance when hydrogen at a specific concentration is introduced into the test chamber. The response time (t_res_) corresponds to the duration required for the signal to reach 90% of its total change upon hydrogen exposure, while the recovery time (t_rec_) represents the time needed for the signal to return to 90% of its baseline value after hydrogen removal.

To ensure precise and reproducible gas sensing measurements, a vacuum-assisted gas-switching system was employed [39]. As illustrated in Supplementary Figure S1a, the setup consists of two connected vacuum chambers: a gas-mixing chamber (≈4.26 L) and a testing chamber (≈0.96 L). During each measurement cycle, the gas-mixing chamber was first evacuated and then filled with hydrogen and synthetic air according to the required partial pressures to obtain the desired hydrogen concentration. The testing chamber was similarly evacuated before exposure. Once the valve between the two chambers was opened, the pre-mixed gas rapidly entered the testing chamber, achieving full gas exchange within seconds. To maintain consistent pressure, the total pressure in the gas-mixing chamber was controlled at 1.23 atm, resulting in an equilibrium pressure of 1 atm after both chambers were connected. Following the sensing step, the testing chamber was re-evacuated, and high-purity air (1 atm) was introduced through a precision pressure regulator to enable the recovery process. This vacuum-assisted gas replacement method ensured highly uniform test conditions and stable, repeatable sensor responses.

## 3. Results and Discussion

### 3.1. Structure of WO_3_ Films

**Figure 2:**
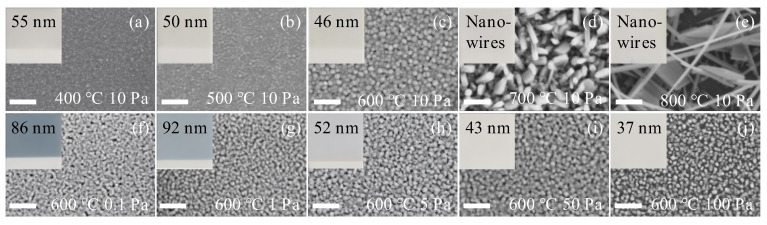
Scanning electron micrographs of WO_3_ thin films deposited at different substrate temperatures (400–800 °C) and oxygen partial pressures. (**a**–**e**) WO_3_ thin films deposited at different substrate temperatures under a fixed oxygen partial pressure; (**f**–**j**) WO_3_ thin films deposited at 600 °C under various oxygen partial pressures. The detailed preparation conditions for each sample are indicated within the corresponding panels. Optical images are shown as insets. The scale bar corresponds to 200 nm.

To fabricate WO_3_ thin films optimized for surface-enhanced sensitivity investigations, an extensive series of experiments was conducted to systematically vary substrate temperature and oxygen partial pressure during deposition, aiming to identify the most favorable conditions. Initially, the oxygen partial pressure was fixed at 10 Pa, while depositions were performed at substrate temperatures in the range of 400–800 °C. Figure 2a–e present the corresponding optical and scanning electron micrographs, alongside thickness measurements at each temperature. The progressive lightening of the film colour with increasing substrate temperature is attributed to the reduction in film thickness, which weakens optical absorption. The SEM images reveal that at 400–600 °C the films retain a continuous morphology, whereas at 700–800 °C the enhanced surface diffusivity of adatoms and re-evaporation of volatile species promote grain coalescence and anisotropic growth, leading to nanorod- and nanosheet-like structures and ultimately a discontinuous network. This morphological transition accounts for the loss of preferred crystallographic orientation and the complete optical transparency observed at the highest temperatures. Thickness measurements obtained via step profiling indicated that films deposited at 400–600 °C possessed thicknesses ranging from 46 to 55 nm; however, measurements for the 700 °C and 800 °C samples were characterized by significant noise, complicating precise thickness determination.

**Figure 3:**
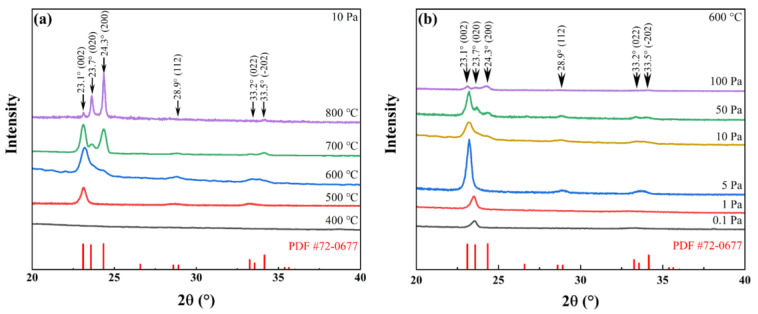
XRD patterns of WO_3_ sample. (**a**) Samples were deposited at varying substrate temperatures while maintaining an oxygen partial pressure of 10 Pa; (**b**) Samples were deposited at a constant temperature of 600 °C under different oxygen partial pressures.

To elucidate the surface morphology and microstructural evolution, SEM analysis was employed. As depicted in Figure 2a–e, films deposited at 400 °C and 500 °C exhibited relatively smooth surfaces with faintly discernible thin film particles. Increasing the substrate temperature to 600 °C resulted in a rougher surface texture, with more prominent particles whose average grain size increased from ~20 nm at 500 °C to ~30 nm at 600 °C. Quantitative analysis further reveals that the average grain size increases from ~15 nm at 400 °C to 20 nm at 500 °C, 30 nm at 600 °C, and evolves into ~50 nm nanorods at 700 °C. At 800 °C, the WO_3_ films transform into a heterogeneous mixture of slender nanowires (∼25 nm in diameter) and nanosheets (∼200 nm in lateral dimension). The influence of oxygen partial pressure on particle size is comparatively weaker. At a constant substrate temperature of 600 °C, the WO_3_ grain size gradually increases from ~20 nm at 0.1–1 Pa to ~25 nm at 5 Pa, and stabilizes around 30 nm at pressures above 10 Pa. This observation indicates that substrate temperature plays a dominant role in determining crystallinity and grain coalescence, as corroborated by the enhanced diffraction intensity in Figure 3a. In contrast, oxygen pressure mainly regulates the kinetic energy of ablated species during PLD; at low pressures, energetic particles (~100 eV) bombard the film surface, hindering crystal growth and leading to smaller grains and partial oxygen deficiency.

XRD analysis (Figure 3a) demonstrates that increasing the substrate temperature induces a progressive transformation of the film from an amorphous phase at 400 °C to a preferentially oriented crystalline phase at 500 °C and 600 °C, accompanied by a marked enhancement in crystallinity. However, further elevation of the substrate temperature, which leads to the formation of nanowires or nanosheets, results in a loss of crystal orientation order and presents challenges in morphological control. To accurately assess the optical and electrical responses of tungsten oxide films to hydrogen exposure, it is essential that the sample morphology be controllable, surface roughness be moderate, and crystallinity be well-defined [41]. Considering these factors, a deposition temperature of 600 °C was selected. Subsequently, tungsten oxide films were deposited under varying oxygen partial pressures of 0.1 Pa, 1 Pa, 5 Pa, 10 Pa, 50 Pa, and 100 Pa to investigate the influence of oxygen pressure on WO_3_ film properties and to identify optimal deposition conditions.

Figure 2f–j present optical photographs, scanning electron micrographs, and thickness measurements of WO_3_ films fabricated under different oxygen pressures during deposition. The optical images reveal that the sample exhibits a dark blue coloration at an oxygen pressure of 0.1 Pa. With increasing oxygen pressure, the film color transitions progressively to light blue (1 Pa), light purple (5 Pa), and ultimately becomes transparent at higher pressures (50 Pa and 100 Pa). This observation aligns with existing literature [42,43,44], which reports that oxygen-deficient tungsten oxide samples display various shades of blue, with increasing oxygen content leading to a gradual lightening of color until transparency is achieved. The samples prepared in this study conform to this trend, as the increasing oxygen partial pressure correlates with enhanced transparency of the WO_3_ films, indicative of a gradual increase in the tungsten-to-oxygen stoichiometric ratio. Furthermore, XPS analysis (Supplementary Figure S2) confirmed that the oxygen-to-tungsten ratio and the average oxidation state of W gradually increased with higher deposition oxygen pressure, consistent with the optical appearance of the WO_3_ films.

Thickness measurements reveal that the film attains a maximum thickness of 92 nm at an oxygen pressure of 1 Pa, after which the thickness progressively diminishes with further increases in oxygen pressure. This phenomenon can be primarily attributed to two competing effects during deposition: the oxygen pressure facilitates the complete oxidation of the tungsten oxide film—wherein more thorough oxidation correlates with increased film thickness—while simultaneously inducing scattering of the deposition particles enroute from the target to the substrate. Elevated oxygen partial pressure intensifies this scattering, thereby reducing the number of particles that successfully reach the substrate and consequently yielding a thinner film. Thus, the interplay between the oxidation process, which promotes thickness growth, and the scattering effect, which diminishes it, culminates in the observed maximum film thickness at 1 Pa oxygen pressure. SEM analyses (Figure 2f–j) demonstrate that all samples deposited at 600 °C comprise fine particulate structures, corroborating the findings from subsequent XRD assessments.

**Figure 4:**
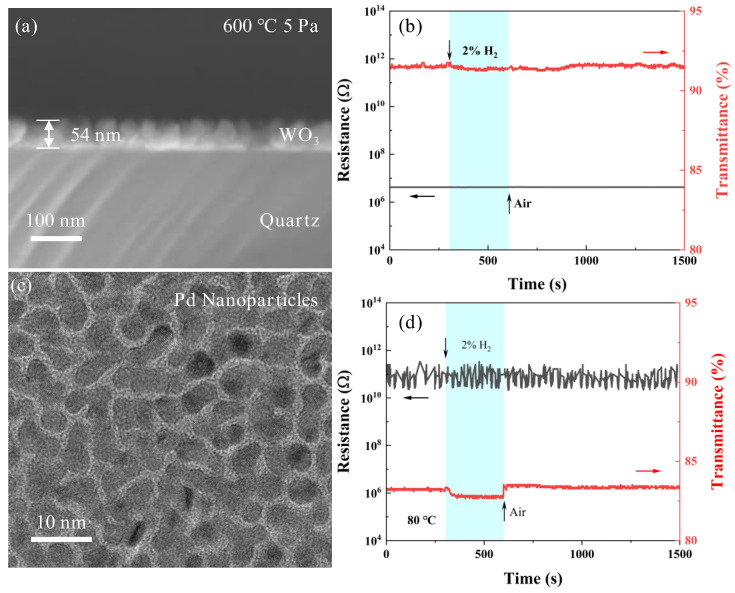
(**a**,**b**) Cross-sectional scanning electron micrograph of WO_3_ film synthesized at 600 °C under an oxygen partial pressure of 5 Pa, accompanied by their corresponding optical and electrical responses upon exposure to 2% *v*/*v* H_2_ gas; (**c**,**d**) Transmission electron micrograph of Pd films with an approximate thickness of 3 nm, together with their optical and electrical responses to 2% *v*/*v* H_2_ gas.

Figure 3b presents the XRD patterns of samples synthesized at 600 °C under varying oxygen pressure. Comparison with reference data (PDF#72-0677) confirms that the films correspond to the monoclinic phase of WO_3_, which is typically observed at ambient temperature. The sample prepared at an oxygen pressure of 0.1 Pa demonstrates a low degree of oxidation, as evidenced by a weak diffraction peak at the (020) crystallographic plane. With increasing oxygen pressure, the tungsten-to-oxygen ratio in the samples progressively increases, accompanied by an enhancement in crystallinity. Notably, the preferred crystallographic orientation transitions from the (020) plane at 1 Pa to the (002) plane at 5 Pa. In addition, the sample fabricated at 600 °C and 5 Pa exhibits the most pronounced preferred orientation, as evidenced by the highest diffraction peak intensity. The cross-sectional scanning electron micrograph depicted in Figure 4a reveals that this sample features highly uniform vertically aligned crystalline structure on the substrate, which accounts for its optimal preferred orientation. When the oxygen pressure surpasses 10 Pa, the elevated gas pressure results in a gradual reduction in both the film deposition rate and the kinetic energy of the depositing species, leading to a more random grain arrangement and a loss of preferred orientation. Specifically, the sample prepared at 100 Pa is notably thin, which corresponds to low-intensity XRD diffraction peaks.

This study primarily aims to investigate the surface and interface sensitization mechanisms of WO_3_ thin films. After obtaining the WO_3_ film structure suitable for surface modification studies, further optimization was guided by our previous findings [45], which showed that samples with slight oxygen deficiency tend to exhibit superior performance compared to strictly stoichiometric compositions. Based on the comprehensive structural and compositional analyses described above, the WO_3_ film deposited at 600 °C under an oxygen pressure of 5 Pa was selected as the optimal sample for subsequent studies. This film exhibits high crystallinity with vertically oriented grains that provide nanoscale pathways facilitating gas diffusion. The measured stoichiometry (WO_2.77_) corresponds to a moderate concentration of oxygen vacancies, which is beneficial for hydrogen adsorption and reaction kinetics. Additionally, the relatively rough surface morphology promotes uniform dispersion of catalytic noble metals such as Pd and Pt. Therefore, all surface functionalization and sensing experiments presented in this work were performed using WO_3_ films prepared under these optimized deposition parameters.

### 3.2. Metal-Functionalized Samples

Prior to commencing the investigation of surface sensitization, the hydrogen sensitivity of WO_3_ thin films fabricated at 600 °C and 5 Pa without any surface modification was evaluated. The findings indicated that at 80 °C (Figure 4b), the WO_3_ thin films did not exhibit any optical or electrical response upon exposure to hydrogen gas. To clarify the origin of hydrogen sensing enhancement in the Pd/WO_3_ system, a nominally 3 nm thick palladium (Pd) film was also prepared and tested separately as a control sample. Figure 4c illustrates the morphology of the 3 nm Pd film deposited onto a TEM-specific copper grid coated with an ultrathin amorphous carbon film, which serves as a stable and conductive support for nanoscale imaging. The Pd nanoparticles were observed to be uniformly distributed in an island-like configuration across the smooth amorphous carbon surface, with particle sizes ranging from approximately 3 to 5 nm. To elucidate the intrinsic hydrogen response of the Pd catalytic material, a 3-nm Pd film was deposited on a quartz substrate, and its electrical and optical responses to a 2% *v*/*v* H_2_/air mixture were measured. As depicted in Figure 4d, the electrical resistance of the Pd film remained unresponsive to hydrogen exposure, fluctuating around 10^11^ Ω. This lack of electrical response is attributed to the relatively large interparticle distances between adjacent Pd nano-islands, which prevent resistivity changes induced by hydrogen absorption from influencing the overall film resistance. Conversely, a 0.5% decrease in optical transmittance was observed, likely resulting from hydrogen absorption-induced expansion of Pd nanoparticles, which reduces the inter-island gaps [46,47]. It is anticipated that when a 3-nm Pd layer is deposited onto the comparatively rougher surface of WO_3_ thin films, as opposed to quartz substrates, the dispersion of Pd nanoparticles will increase further, thereby diminishing the contribution of the intrinsic hydrogen response of Pd to the overall device performance. Additionally, other modification materials with a thickness of 3 nm exhibited no hydrogen response at 80 °C.

**Figure 5:**
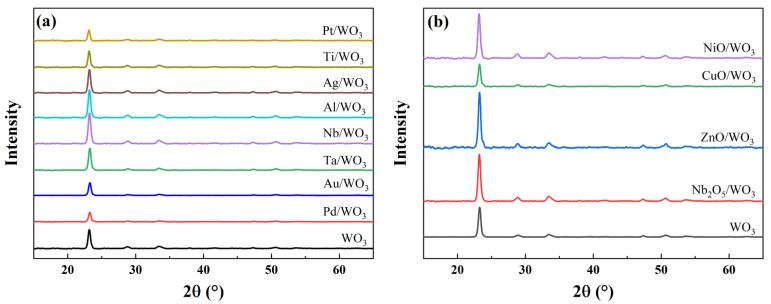
XRD patterns of WO_3_ thin films featuring various surface modification layers: (**a**) metallic coatings, (**b**) oxide coatings.

Figure 5a,b depict the XRD patterns of WO_3_ films modified with the indicated materials. The unmodified WO_3_ film exhibits a prominent diffraction peak at 2θ = 23.17°, corresponding to the (002) plane of monoclinic WO_3_, in agreement with the standard pattern (PDF#72-0677). After modification with different metallic or oxide layers, the position and intensity of this characteristic peak remain essentially unchanged, indicating that the crystalline structure and preferred orientation of the WO_3_ films are largely preserved. No additional diffraction peaks attributable to the surface modification materials were detected, likely because the deposited layer thickness (3 nm) is below the XRD detection limit. Supplementary Figure S3 displays the atomic force micrographs of both the pristine WO_3_ film and the WO_3_ film modified with Pd. The pristine WO_3_ film consists of nanoparticles of ~35 nm in average diameter, resulting in a smooth surface. Following modification with a 3 nm Pd layer, the surface morphology retains the nanoparticulate structure; however, numerous nanoscale protrusions are observed atop the particles. This morphological alteration provides evidence for the successful deposition of Pd onto the WO_3_ film surface.

**Figure 6:**
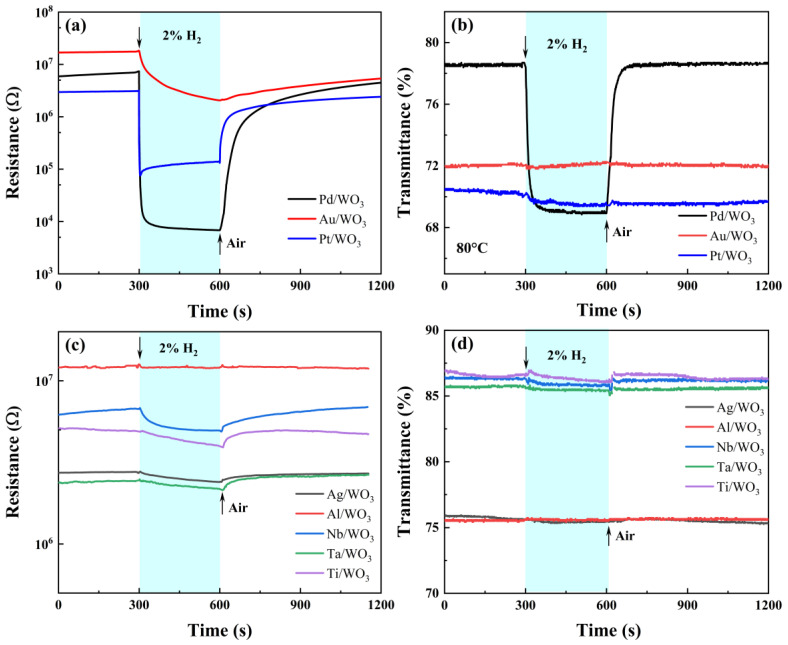
Optical and electrical response characteristics of WO_3_ decorated with (**a**,**b**) Pd, Au, and Pt, and (**c**,**d**) Ag, Al, Nb, Ti, and Ta under 2% *v*/*v* H_2_ at 80 °C.

Figure 6 presents the electrical (DC resistance) and optical (transmittance at 785 nm) response profiles of WO_3_ films modified with two distinct groups of metallic materials treated by 2% *v*/*v* H_2_ gas. The parameters of response magnitude, t_res_, and t_rec_ are summarized in Table 1 and Table 2, with all measurements conducted at a controlled temperature of 80 °C. Taking the Pd-modified WO_3_ film as an example (black trace in Figure 6a), the resistance remains almost constant in synthetic air, then drops by nearly three orders of magnitude (from 6.993 × 10^6^ Ω to 6.833 × 10^3^ Ω) immediately after 2% *v*/*v* H_2_ is introduced, and finally recovers close to its initial value within 770 s after hydrogen is purged. Similar behaviour is observed for Pt- and Au-modified samples, but with markedly different response amplitudes and time constants. In terms of both the resistance and the optical responses, the first group of metals (Figure 6a,b, Pd, Pt, Au) demonstrates a at least six times greater response magnitude compared to the second group (Figure 6c,d, Ag, Al, Nb, Ti, Ta). Within the first group, substantial variability in response magnitude is observed. Notably, Pd exhibits the most pronounced electrical response, achieving a response value of 1022 (Figure 6a), which is approximately 45 times greater than that of Pt and 122 times greater than that of Au.

In the metal-modified samples, the work-function difference between the metal and WO_3_ establishes a surface depletion or accumulation layer at the interface. Meanwhile, upon exposure to hydrogen, catalytic dissociation of H_2_ on active metals such as Pd generates atomic hydrogen that spills over onto WO_3_, where it reacts with adsorbed oxygen species and is incorporated into the lattice. These processes increase the electron concentration in WO_3_ and reduce the width of the surface depletion region, leading to a pronounced decrease in resistance. At the same time, the formation of hydrogen tungsten bronze H_x_WO_3_ introduces donor levels near the conduction band and enables intervalence charge transfer between W^5+^/W^6+^ sites, resulting in additional visible-light absorption and a reduction in transmittance (gasochromic effect). Thus, the combined electronic and structural modifications induced by hydrogen explain the observed changes in both resistance and transmittance, with the electrical response depending on both the metal work function and catalytic activity, while the optical response is mainly governed by the catalytic ability of different metals.

Given that the work functions of Pd (5.12 eV) and Au (5.10 eV) are nearly identical [31], and Au exhibits negligible catalytic activity toward hydrogen and oxygen, the enhanced sensitisation for the Au-modified sample can be primarily attributed to the electronic sensitization effect. As depicted in Figure 6a,b the difference in response magnitude between Pd- and Au-modified samples therefore mainly originates from the catalytic sensitization effect introduced by Pd. In contrast, when comparing Pd- and Pt-modified samples, the significantly lower work function of Pd compared to Pt (5.65 eV) would suggest a weaker purely electronic sensitisation effect. However, the Pd-modified sample still exhibits an electrical response amplitude ~45 times higher than that of Pt, indicating that the catalytic activity of Pd dominates the overall sensing behaviour. This conclusion is further supported by the optical response results (Figure 6b and Table 2), where only the Pd-modified sample demonstrates a distinct transmittance change accompanied by the characteristic blue coloration of WO_3_, indicative of a gasochromic effect associated with tungsten bronze (H_x_WO_3_) formation.

As shown in Figure 6b, only the Pd-modified WO_3_ film exhibited a pronounced optical response to hydrogen exposure. This phenomenon arises from the gasochromic reaction facilitated by Pd catalysis [48,49]. Molecular hydrogen (H_2_) is first dissociated on the Pd surface into atomic hydrogen (Equation (1)), which then migrates to the WO_3_ surface and diffuses into its lattice (Equation (2)). The incorporated hydrogen atoms reduce W^6+^ to W^5+^, forming hydrogen tungsten bronze (H_x_WO_3_) according to Equation (3):

(1) $$H_{2}⟷\;2H_{\mathrm{atom}}$$

(2) $$H_{\mathrm{atom}}⟷\;H^{+}+e^{-}$$

(3) $$xH^{+}+xe^{-}+W^{6+}O_{3}^{2-}\;⟷\;{\;H}_{x}W_{x}^{5+}W_{1-x}^{6+}O_{3}^{2-}\left(\mathrm{Tungsten}\;\mathrm{bronze}\right)$$

The formation of H_x_WO_3_ introduces donor levels near the conduction band and leads to intervalence charge transfer between W^5+^ and W^6+^ sites, resulting in visible-light absorption and a characteristic blue coloration. As shown in Supplementary Figure S4, the optical transmittance of WO_3_ films deposited at 600 °C increases with oxygen partial pressure. Films prepared at 0.1 and 1 Pa exhibit strong absorption above 400 nm, while those prepared at ≥10 Pa display high transparency across the visible range, consistent with their visual color and XPS-determined O:W ratio (Supplementary Figure S2). The corresponding optical bandgap values (2.62–3.02 eV, Supplementary Figure S5) align well with literature data [21,50] and reflect the combined influence of stoichiometry, crystallinity, and film thickness on the optical properties. In contrast, other metal modifiers such as Pt, Au, and Ag, despite having comparable work functions, lack sufficient catalytic activity for hydrogen dissociation, and thus no observable optical response was detected. In contrast, other metal modifiers such as Pt, Au, and Ag, despite having comparable work functions, lack sufficient catalytic activity for hydrogen dissociation, and thus no observable optical response was detected.

The present analysis centers on the second group of metallic materials, specifically Ag, Al, Nb, Ti, and Ta. As illustrated in Figure 6c,d, modification of WO_3_ with these five metals results in generally weak electrical response amplitudes with minimal variation, and the material continues to exhibit no observable optical response. Several factors account for this phenomenon. Firstly, from an electronic perspective, the work functions of the metals in this group are approximately uniform, around 4.3 eV, and are considerably lower than those of the metals in the first group. Secondly, regarding catalytic activity, these metals typically demonstrate negligible or no catalytic effects. The minor differences in response amplitude observed among Nb, Ti, Ag, and Ta may be attributed to slight variations in their catalytic properties.

For the three samples exhibiting larger response amplitudes (Pd, Au, and Pt), the recovery times were correspondingly longer. Among them, Pt and Pd showed shorter recovery times than Au, which can be attributed to their catalytic activity toward both hydrogen and oxygen [27]. In contrast, the second group of metals with lower work functions exhibited smaller response amplitudes and shorter recovery times, possibly because the re-adsorption of oxygen ions to equilibrium occurs more rapidly.

In summary, with respect to the sensitization of WO_3_ via metal modification at the current testing temperature of 80 °C, Pd uniquely exhibits both electronic and catalytic sensitization effects, with the catalytic contribution yielding a substantially greater enhancement. Consequently, WO_3_ samples modified with Pd display electrical and optical hydrogen responses that are significantly superior to those modified with other metals. For the remaining metals, an increase in work function correlates with a stronger electronic sensitization effect.

**Figure 7:**
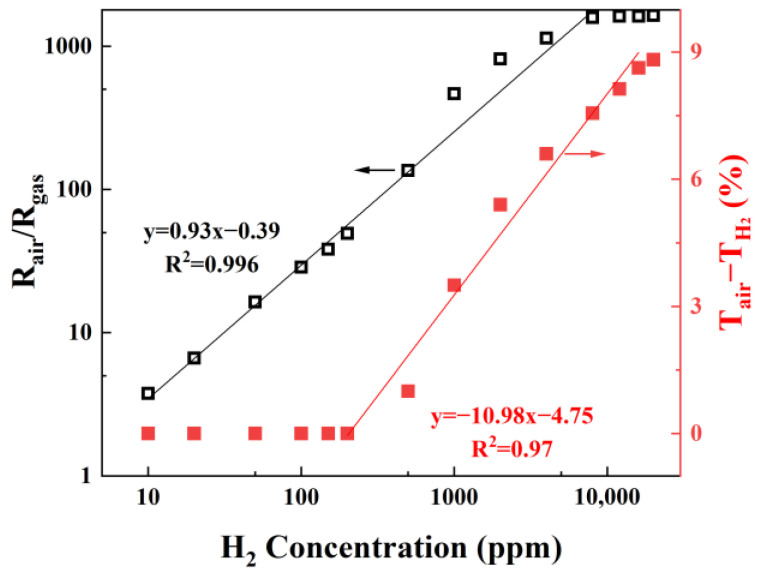
Calibration plots of electrical (R_air_/R_H2_) and optical (T_air_ − T_H2_) responses of Pd/WO_3_ film as a function of hydrogen concentration.

Having established the superior hydrogen sensing behavior of the Pd-modified WO_3_ film, its quantitative response to varying hydrogen concentrations was further examined. Supplementary Figure S6 presents the electrical and optical response curves of the Pd/WO_3_ film to hydrogen concentrations ranging from 10 ppm to 20,000 ppm, the calculated response magnitudes are shown in Figure 7. As shown in these figures, the electrical response remains highly sensitive even at a low concentration of 10 ppm, exhibiting a response magnitude of 3.74, but gradually approaches saturation as the hydrogen concentration nears 10,000 ppm. In contrast, the optical response of the Pd/WO_3_ film is negligible below 500 ppm H_2_ but increases monotonically with higher concentrations, displaying an approximately linear relationship. For safety considerations, hydrogen concentrations above 20,000 ppm were not tested; however, the optical response in this range remains unsaturated, indicating the potential for further extension of the detection range. Therefore, by combining the rapid, low-concentration sensitivity of the electrical signal with the excellent linearity and wide detection range of the optical response, a hybrid optoelectronic hydrogen sensor based on WO_3_ can be envisioned to achieve comprehensive monitoring over a broad concentration span.

To further evaluate the repeatability of the Pd/WO_3_ sensor, cyclic hydrogen exposure tests were carried out under identical operating conditions. As shown in Supplementary Figure S7, the sensor was exposed to 2% *v*/*v* H_2_ for 5 min per cycle, followed by recovery in air. After 20 successive response cycles, both the upper and lower limits of the electrical resistance and optical transmittance remained nearly unchanged, and the response amplitude showed no observable decay. These results confirm the excellent repeatability and stability of the Pd/WO_3_ sensing system.

In addition, to evaluate the selectivity of the Pd-modified WO_3_ sensor, additional measurements were conducted under identical conditions (80 °C, 2% *v*/*v* gas concentration). As shown in Supplementary Figure S8, the sensor exhibited a strong electrical response to 2% *v*/*v* H_2_, while the responses to other gases such as CO, H_2_S, CH_4_, and NH_3_ were negligible. No detectable optical response was observed for these interfering gases. This high selectivity arises from the unique catalytic role of Pd, which facilitates the dissociation of H_2_ molecules into atomic hydrogen and enhances surface reactions at low temperature. In contrast, other gases require elevated temperatures (typically above 200 °C) to achieve comparable reactivity, confirming that the Pd/WO_3_ sensor possesses excellent hydrogen selectivity at 80 °C. These findings confirm that Pd not only enhances the sensitivity but also ensures remarkable selectivity toward hydrogen among various tested gases. As shown in Supplementary Figure S9, the resistance of the Pd/WO_3_ sample in air decreases with increasing relative humidity, indicating that water molecules act as electron donors by adsorbing on the surface either in molecular form or as hydroxyl groups (OH^−^). These species can replace pre-adsorbed oxygen ions (O^−^) and release free electrons, leading to a reduction in electrical resistance. Consequently, the electrical performance degrades significantly at higher humidity levels. In addition, water molecules can interact with Pd, dissociating into hydrogen ions and hydroxyl groups. The formation of hydroxyl species causes partial poisoning of Pd active sites, thereby reducing both the optical and electrical responses of the Pd/WO_3_ sensor toward hydrogen.

### 3.3. MO_x_-Functionalized Samples

**Figure 8:**
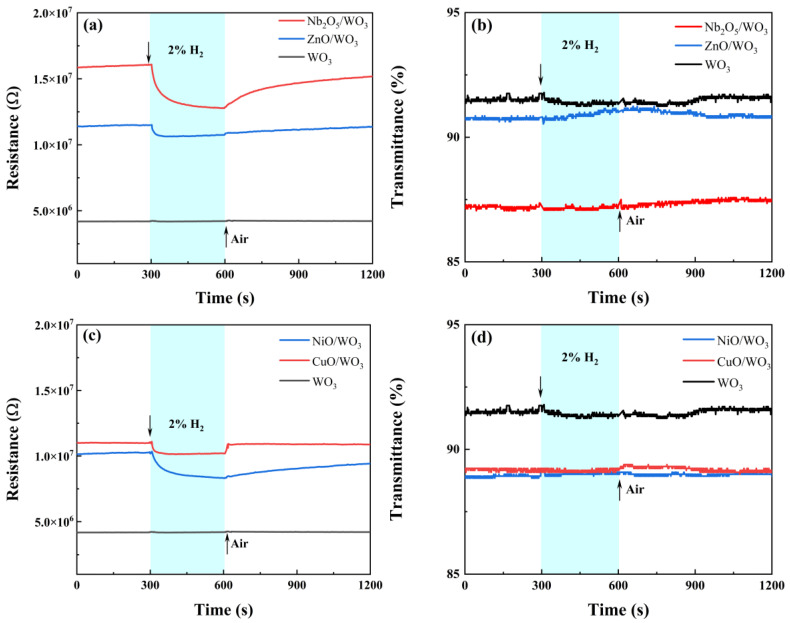
Optical and electrical response characteristics of (**a**,**b**) Nb_2_O_5_ and ZnO-modified WO_3_; (**c**,**d**) NiO and CuO-modified WO_3_ under 2% *v*/*v* H_2_ at 80 °C.

In this study, two n-type MO_x_ semiconductors (Nb_2_O_5_ and ZnO) and two p-type oxide semiconductors (CuO and NiO) were selected to modify the surface of WO_3_ thin films, each with a nominal thickness of 3 nm, in order to investigate the regulatory influence of oxide semiconductor heterojunctions on the hydrogen sensing performance of WO_3_. Figure 8 presents the resistance and transmittance response curves of the MO_x_-modified WO_3_ films when exposed to 2% *v*/*v* H_2_ gas. The corresponding response metrics, including t_res_ and t_rec_, are summarized in Table 3. For comparative purposes, the response curve of the unmodified WO_3_ film to 2% *v*/*v* H_2_, previously shown in Figure 4b, is also included in Figure 8.

Firstly, the MO_x_ modified WO_3_ films did not demonstrate a significant optical response, suggesting that the catalytic activity of these four oxide materials toward hydrogen is either weak or absent. Secondly, given that pure WO_3_ exhibits no response to hydrogen, the observed variations in response magnitude among the four modified samples are primarily ascribed to electronic sensitization effects. Moreover, irrespective of the oxide modifier type (n-type or p-type), those materials exhibiting a larger work function disparity relative to WO_3_ (namely Nb_2_O_5_ and CuO) displayed comparatively higher resistance response magnitudes (1.26 and 1.24, respectively). In contrast, the two modifiers with work functions closely aligned to that of WO_3_ (ZnO and NiO) showed diminished responses. Consequently, the n-n and p-n sensitization effects induced by oxide modification predominantly depend on electronic sensitization mechanisms, with the sensitization effect intensifying as the work function difference relative to WO_3_ increases.

## 4. Conclusions

This study investigated the influence of deposition parameters, including substrate temperature ranging from 400 °C to 800 °C, and oxygen partial pressure between 0.1 Pa and 100 Pa, on the appearance, morphology, and crystallinity of WO_3_ thin films fabricated via PLD. WO_3_ thin films with vertically aligned crystallites synthesized at 600 °C and 5 Pa—conditions that yield strong preferred orientation, moderate oxygen vacancy concentration and favourable morphology for gas diffusion—were selected for surface-modification sensitisation experiments. Among eight different metal modifiers tested, Pd exhibited a markedly enhanced resistance response (S_e_ = 1022) compared to other metals, attributable to its combined catalytic and electronic effects, and also demonstrated a pronounced optical response (S_o_ = 9.7%). In contrast, metals such as Pt, Au, and Ag did not elicit significant optical responses, suggesting that under the present experimental conditions, catalytic sensitization effects were minimal. Consequently, the sensitization effect of metal modifiers primarily depended on electronic sensitization, governed by the work function of metal, with higher work function correlated with more pronounced sensitisation. Pt, possessing the highest work function among the metals studied, exhibited the most effective sensitization. Based on these findings, the developed WO_3_-based hydrogen sensors can be classified as photoelectrically coupled sensors capable of detecting both optical and electrical responses. Pd-modified WO_3_ is more suitable for photoelectric dual-mode sensors or optical hydrogen leak detectors, providing a broad detection range and enhanced safety. In contrast, Pt-modified WO_3_ is ideal for rapid-response hydrogen leak detection applications due to its ultra-fast response time of approximately 0.5 s. In addition, as Pt did not induce gas-related color changes, the sensing mechanism of WO_3_ toward hydrogen was confined to surface interactions, resulting in a rapid response time of 0.5 s, highlighting its suitability for hydrogen leak detection applications. Regarding oxide material modification, the sensitization effect was found to increase with the magnitude of the work function difference between the oxide and WO_3_, independent of the charge carrier type.

## Figures and Tables

**Table 1:** Electrical resistance response characteristics of metal-functionalized WO_3_ thin films.

Metal	R_air_ (Ω)	R_H2_ (Ω)	S_e_ (R_air_/R_H2_)	t_res_ (s)	t_rec_ (s)
Pd	6.993 × 10^6^	6.833 × 10^3^	1022	1	770
Au	1.758 × 10^7^	2.094 × 10^6^	8.4	112	806
Pt	3.085 × 10^6^	1.387 × 10^5^	22.4	0.5	632
Ag	2.763 × 10^6^	2.403 × 10^6^	1.15	239	285
Al	1.228 × 10^7^	1.209 × 10^7^	-	-	-
Nb	6.750 × 10^6^	4.960 × 10^6^	1.36	122	409
Ta	2.437 × 10^6^	2.182 × 10^6^	1.12	253	390
Ti	4.881 × 10^6^	3.981 × 10^6^	1.22	258	129

**Table 2:** Transmittance response characteristics of metal-functionalized WO_3_ thin films.

Metal	T_air_ (%)	T_H2_ (%)	S_O_ (T_air_ − T_H2_) (%)	t_res_ (s)	t_rec_ (s)
Pd	78.6	68.9	9.7	28	43

Note: other samples do not demonstrate any optical response.

**Table 3:** Electrical resistance response characteristics of MO_x_-functionalized WO_3_ thin films.

Metal oxide	R_air_ (Ω)	R_H2_ (Ω)	S_E_ (R_air_/R_H2_)	t_res_ (s)	t_rec_ (s)
NiO	1.099 × 10^7^	1.019 × 10^7^	1.08	25	9
CuO	1.031 × 10^7^	8.315 × 10^6^	1.24	150	753
Nb_2_O_5_	1.609 × 10^7^	1.279 × 10^7^	1.26	120	782
ZnO	1.150 × 10^7^	1.073 × 10^7^	1.07	17	838

## Data Availability

The original contributions presented in this study are included in the article/Supplementary Materials. Further inquiries can be directed to the corresponding authors.

## Supplementary Materials

The following supporting information can be downloaded at https://www.mdpi.com/article/10.3390/s25237268/s1, Figure S1: (a) Schematic of the test chamber of gas sensor signal measurement system. (b) Schematic of the sample holder and electrical test probes. (c) Photograph of the sensor sample (6 × 12 mm, electrode spacing 6 mm); Figure S2: X-ray photoelectron spectroscopy analysis of WO_3_ thin films prepared under different oxygen partial pressures. (a) O 1s and (b) W 4f spectra after Ar^+^ etching and charge correction; Figure S3: Three-dimensional atomic force micrographs of WO_3_ films: (a) WO_3_; (b) Pd/WO_3;_ Figure S4: Optical transmittance spectra of WO_3_ films deposited at 600 °C under different oxygen partial pressures (0.1–100 Pa); Figure S5: Tauc plots of (αhν)^1^^/^^2^ versus photon energy (hν) for WO_3_ films deposited at 600 °C under different oxygen partial pressures: (a) 0.1 Pa, (b) 1 Pa, (c) 5 Pa, (d) 10 Pa, (e) 50 Pa, and (f) 100 Pa; Figure S6: Electrical and optical response characteristics of the Pd-modified WO_3_ film toward different hydrogen concentrations at 80 °C. (a), (b) Dynamic electrical (black) and optical (red) response curves to 10 ppm–2 % H_2_; Figure S7: Repeatability performance of the Pd/WO_3_ thin-film hydrogen sensor at 80 °C under 2 % H_2_. (a) Electrical-resistance response during twenty consecutive hydrogen exposure–recovery cycles (5 min per cycle). (b) Corresponding optical-transmittance variation; Figure S8: Selectivity performance of the Pd-modified WO_3_ sensor toward various gases at 80 °C under 20,000 ppm concentration. (a) Electrical response (×100) and (b) optical response (%); Figure S9: Influence of relative humidity (RH) on the hydrogen sensing performance of the Pd-modified WO_3_ film at 80 °C; Table S1: Process parameters and deposition rates of various metals via magnetron sputtering; Table S2: Process parameters and deposition rates of various MO_x_ via magnetron sputtering.
