# Supplementary material for: Investigation of Surface Modification Effects on the Optical and Electrical Hydrogen Sensing Characteristics of WO3 Films
Source: Sensors (Basel). 2025 Nov 28;25(23):7268. doi: 10.3390/s25237268 (PMC12693903; doi:10.3390/s25237268)
Supplement: Supplementary file 1 [file sensors-25-07268-s001.zip › sensors-3964478-supplementary.pdf]

Supporting Information

Investigation of Surface Modification Effects on the Optical and Electrical Hydrogen Sensing Characteristics of WO_3 Films

Jiabin Hu [1], Jie Wei [2], Jianmin Ye [1], Wen Ye [1,*], Ying Li [3], Zhe Lv [4], and Meng Zhao [1,*]

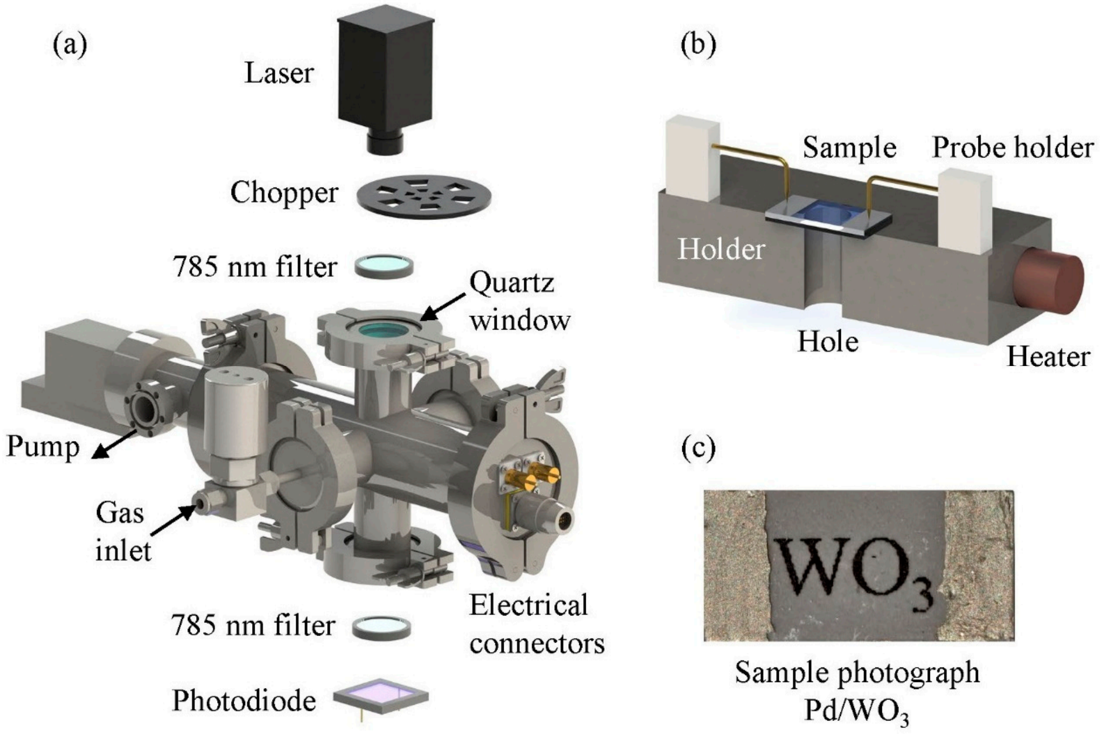

Supplementary Figure S1. (a) Schematic of the test chamber of gas sensor signal measurement system. (b) Schematic of the sample holder and electrical test probes. (c) Photograph of the sensor sample (6×12 mm, electrode spacing 6 mm).

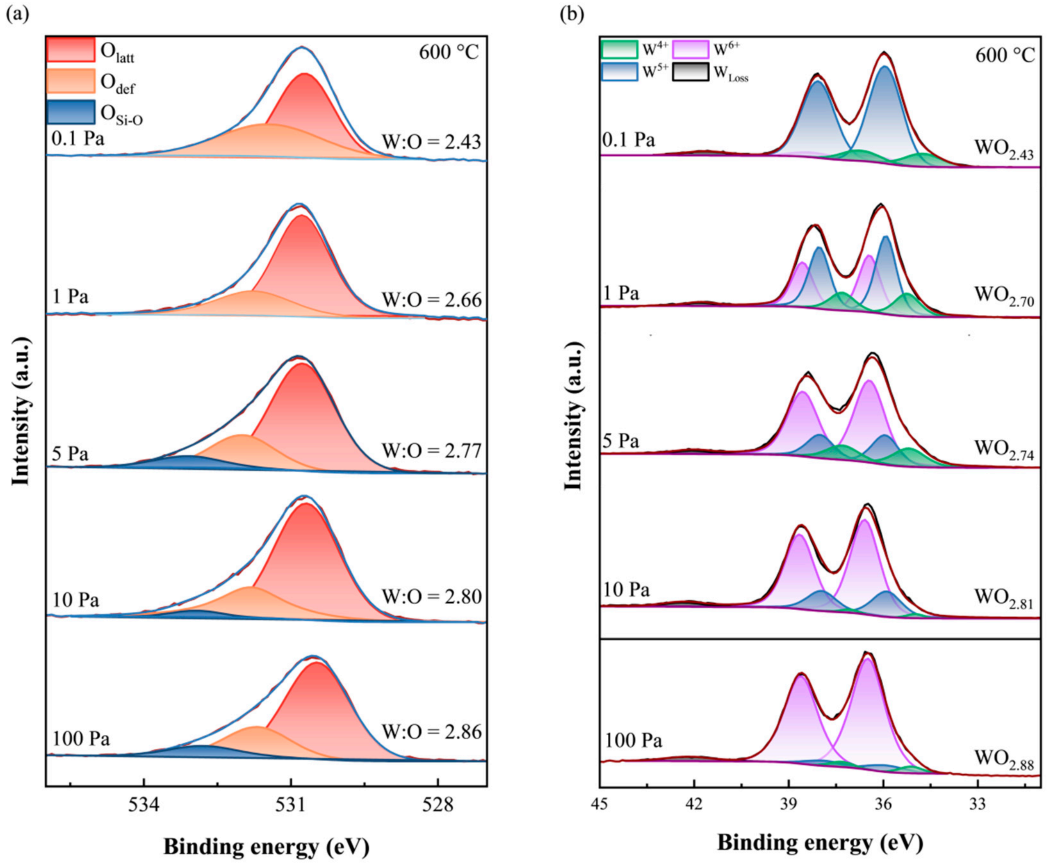

Supplementary Figure S2. X-ray photoelectron spectroscopy analysis of WO_3 thin films prepared under different oxygen partial pressures. (a) O 1s and (b) W 4f spectra after Ar^+ etching and charge correction.

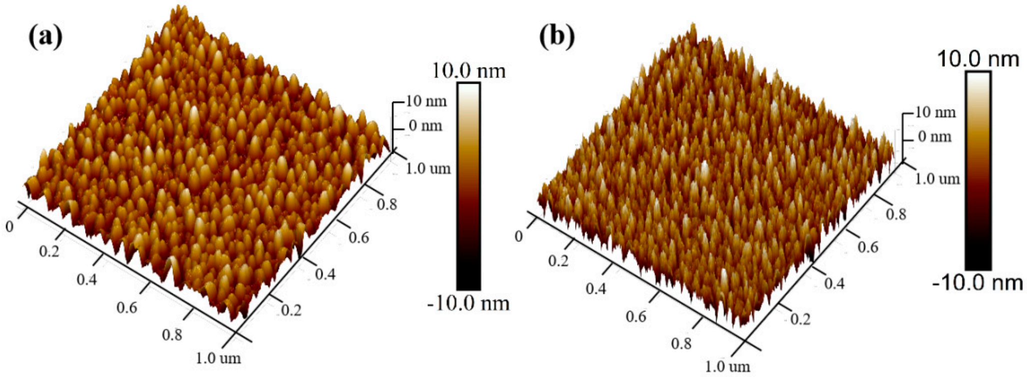

Supplementary Figure S3. Three-dimensional atomic force micrographs of WO_3 films: (**a**) WO_3; (**b**) Pd/WO_3.

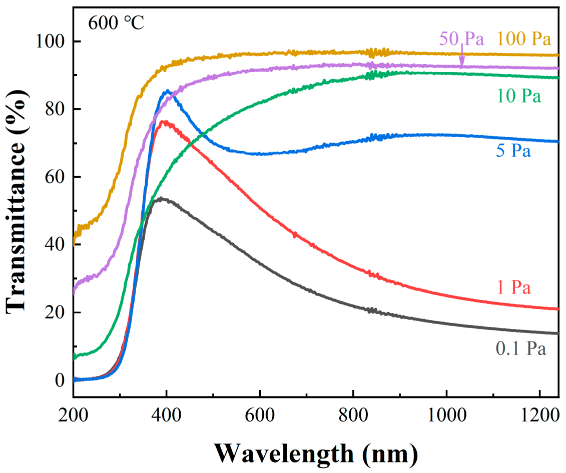

Supplementary Figure S4. Optical transmittance spectra of WO_3 films deposited at 600 °C under different oxygen partial pressures (0.1–100 Pa).

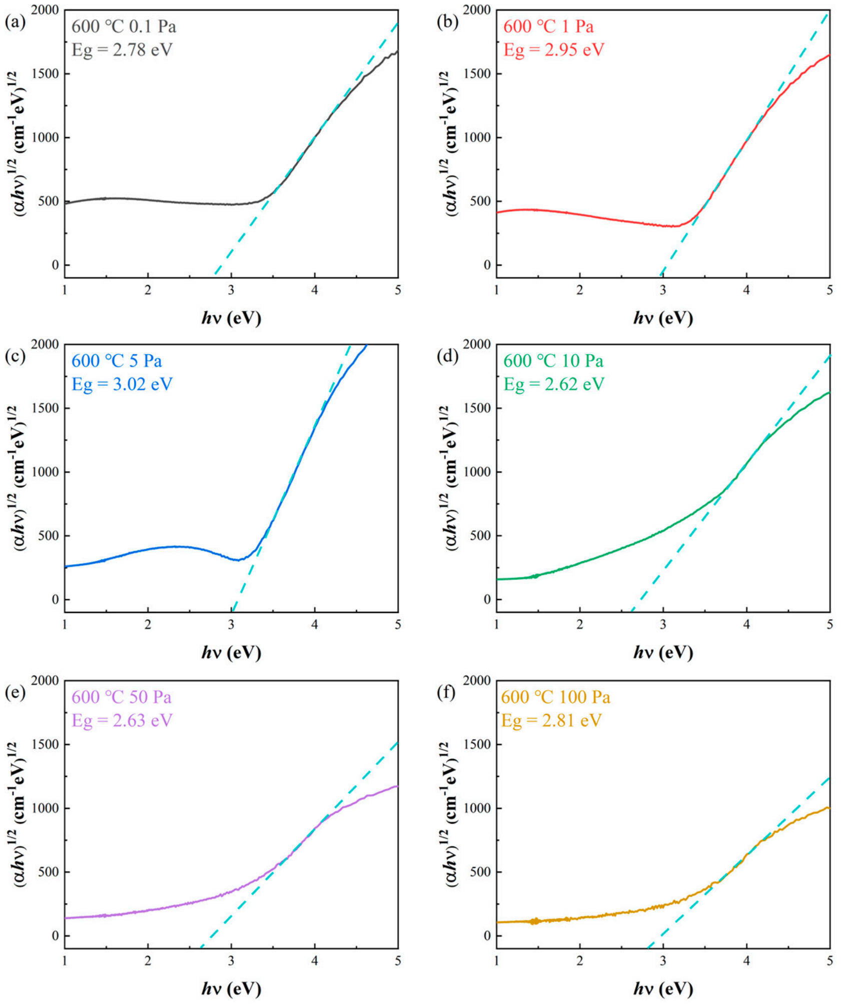

Supplementary Figure S5. Tauc plots of $(\alpha h\nu)^{1/2}$ versus photon energy (hν) for WO_3 films deposited at 600 °C under different oxygen partial pressures: (a) 0.1 Pa, (b) 1 Pa, (c) 5 Pa, (d) 10 Pa, (e) 50 Pa, and (f) 100 Pa.

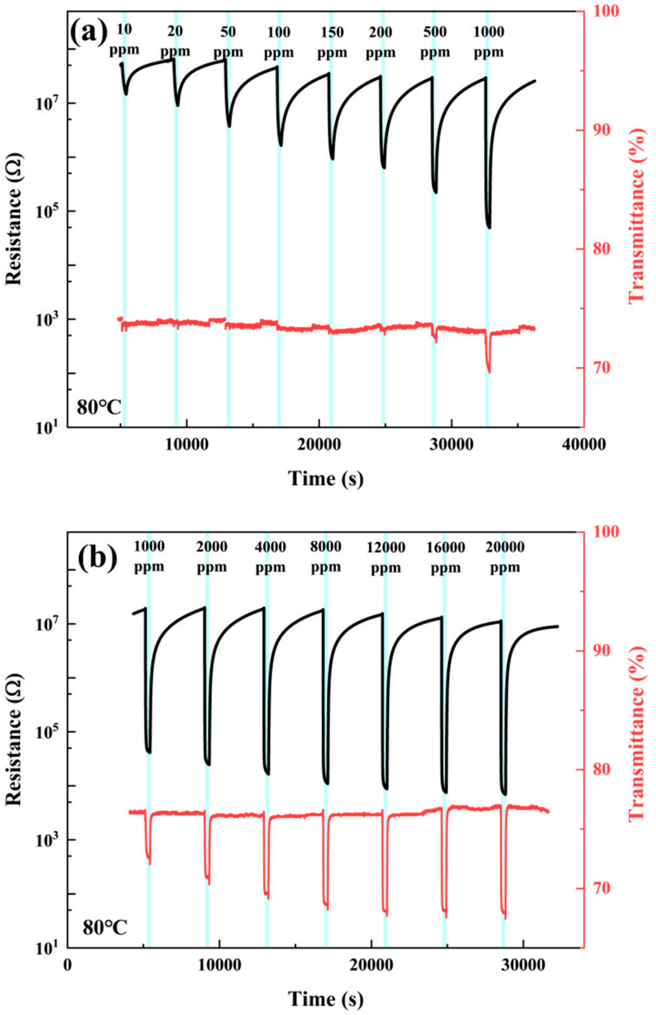

Supplementary Figure S6. Electrical and optical response characteristics of the Pd-modified WO_3 film toward different hydrogen concentrations at 80 °C. (a), (b) Dynamic electrical (black) and optical (red) response curves to 10 ppm–2 % H_2.

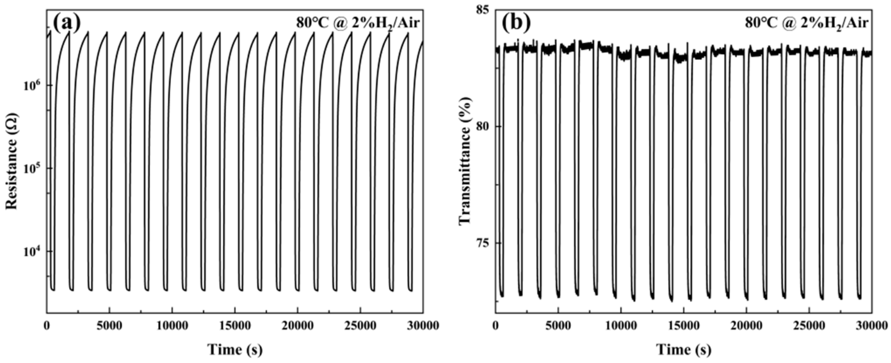

Supplementary Figure S7 Repeatability performance of the Pd/WO_3 thin-film hydrogen sensor at 80 °C under 2 % H_2. (a) Electrical-resistance response during twenty consecutive hydrogen exposure–recovery cycles (5 min per cycle). (b) Corresponding optical-transmittance variation.

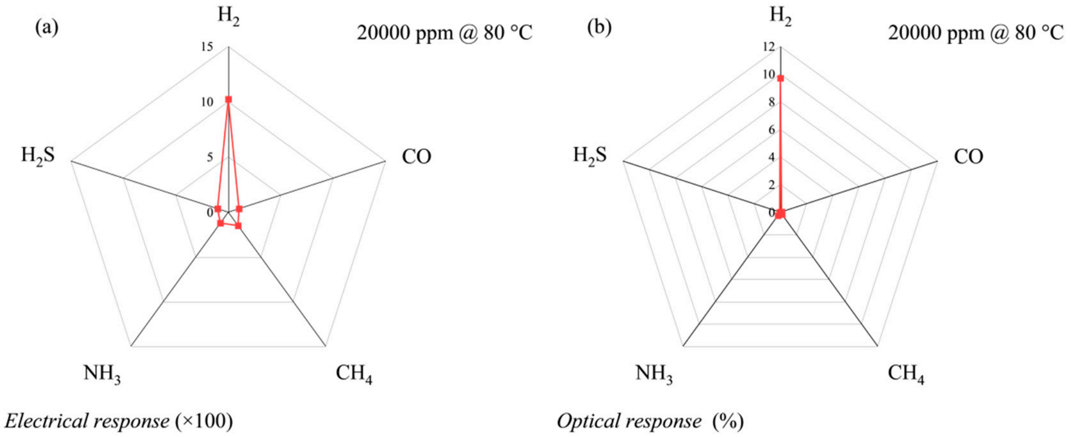

Supplementary Figure S8. Selectivity performance of the Pd-modified WO_3 sensor toward various gases at 80 °C under 20000 ppm concentration. (a) Electrical response (×100) and (b) optical response (%).

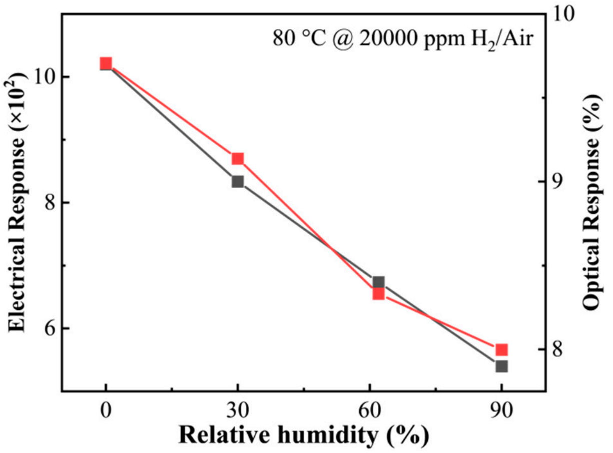

Supplementary Figure S9. Influence of relative humidity (RH) on the hydrogen sensing performance of the Pd-modified WO_3 film at 80 °C.

Supplementary Table S1. Process parameters and deposition rates of various metals via magnetron sputtering

Materials	Pressure (Pa)	Power (W)	Deposition rate (nm/min)
Pd	2	20	3.0
Au	2	20	4.7
Ti	2	100	3.1
Al	2	100	2.7
Nb	2	50	4.1
Ag	2	20	3.7
Ta	2	50	5.7
Pt	2	20	3.0

Supplementary Table S2. Process parameters and deposition rates of various MO_x via magnetron sputtering

Materials	Pressure (Pa)	$Ar:O_2$	Power (W)	Deposition rate (nm/min)
ZnO	1	1:4	200	4.3
Nb_2O_5	1	1:4	180	2.1
CuO	1	1:4	100	3.1
NiO	1	1:4	120	2.9
